# Probiotic effects of *Lactococcus lactis* and *Leuconostoc mesenteroides* on stress and longevity in *Caenorhabditis elegans*

**DOI:** 10.3389/fphys.2023.1207705

**Published:** 2023-09-12

**Authors:** Mylissa A. Stover, Brenda Tinoco-Bravo, Crystal A. Shults, Sydney Marouk, Ratnakar Deole, Jacob R. Manjarrez

**Affiliations:** Biochemistry and Microbiology Department, Oklahoma State University Center for Health Sciences, Tulsa, OK, United States

**Keywords:** *Caenorhabditis elegans*, *Lactococcus lactis*, *Leuconostoc mesenteroides*, probiotic, stress, lifespan, longevity

## Abstract

The short lifespan of *Caenorhabditis elegans* enables the efficient investigation of probiotic interventions affecting stress and longevity involving the potential therapeutic value of *Lactococcus lactis* and *Leuconostoc mesenteroides* isolated from organic basil. The lactic acid bacteria were cultured from the produce collected from a local grocery store in Tulsa, Oklahoma, and then identified through 16S rDNA sequencing and biochemical tests. To dive deep into this analysis for potential probiotic therapy, we used fluorescent reporters that allow us to assess the differential induction of multiple stress pathways such as oxidative stress and the cytoplasmic, endoplasmic reticulum, and the mitochondrial unfolded protein response. This is combined with the classic health span measurements of survival, development, and fecundity, allowing a wide range of organismal observations of the different communities of microbes supported by probiotic supplementation with *Lactococcus lactis* and *Leuconostoc mesenteroides*. These strains were initially assessed in relation to the *Escherichia coli* feeding strain OP50 and the *C. elegans* microbiome. The supplementation showed a reduction in the median lifespan of the worms colonized within the microbiome. This was unsurprising, as negative results are common when probiotics are introduced into healthy microbiomes. To further assess the supplementation potential of these strains on an unhealthy (undifferentiated) microbiome, the typical axenic *C. elegans* diet, OP50, was used to simulate this single-species biome. The addition of lactic acid bacteria to OP50 led to a significant improvement in the median and overall survival in simulated biomes, indicating their potential in probiotic therapy. The study analyzed the supplemented cultures in terms of *C. elegans*’ morphology, locomotor behavior, reproduction, and stress responses, revealing unique characteristics and stress response patterns for each group. As the microbiome’s influence on the health span gains interest, the study aims to understand the microbiome relationships that result in differential stress resistance and lifespans by supplementing microbiomes with *Lactococcus lactis* and *Leuconostoc mesenteroides* isolated from organic basil in *C. elegans*.

## 1 Introduction

There is growing interest in improving the overall health and wellbeing of the gut microbiota by using probiotics as a natural and safe approach, especially in relation to aging and stress (Cryan and Dinan, 2012; O’Toole and Jeffery, 2015; Kim et al., 2017; Ticinesi et al., 2019). The source of probiotic strains is an important factor to consider, as the properties of probiotics can vary depending on the source from which they are isolated (Ray and Didier, 2014; Langkamp-Henken et al., 2015; Kumar et al., 2022; Zhang et al., 2022). Bacteria isolated from organic produce may be a valuable source of probiotics for several reasons. First, organic produce is grown without the use of synthetic fertilizers, pesticides, and other chemicals that may have adverse effects on the microbiota of the produce. This may result in resident microbiota that is more diverse and potentially more beneficial for human health when consumed. Bacteria isolated from organic produce may have unique properties that make them well-suited for use as probiotics. For example, they may have enhanced survival and colonization abilities in the human gut, or they may produce metabolites that have beneficial effects on gut health (Requena et al., 2018; Kumar et al., 2022; Zhang et al., 2022).

However, it is important to note that not all bacteria isolated from organic produce may be suitable for use as probiotics. Proper safety and efficacy assessments should be conducted to ensure that any potential probiotic strains are safe for human consumption and have the desired beneficial effects on gut health. In this study, we investigate the probiotic potential of two strains isolated from Oklahoma-grown organic basil, positively identified through 16S rRNA gene sequencing, *Lactococcus lactis* (*L. lactis*) and *Leuconostoc mesenteroides* (*L. mesenteroides*) (Van Tieghem, 1878; Schleifer et al., 1985).

A well-established model organism that has been used extensively in aging and stress research is *Caenorhabditis elegans* (*C. elegans*) (Lithgow et al., 1995; Gems and Riddle, 2000; Lithgow and Walker, 2002; Panowski and Dillin, 2009; Kenyon, 2010; Gems and Partridge, 2013). One advantage of using *C. elegans* as a model for evaluating probiotic therapy is its short lifespan, which allows for the rapid screening of potential therapeutic interventions (Brenner, 1974; Kumar et al., 2022; Zhang et al., 2022). In addition, *C. elegans* has a well-characterized genome and is genetically tractable (C. elegans Sequencing Consortium, 1998; Howe et al., 2016), making it a useful tool for studying the mechanisms underlying the effects of probiotics on host stress responses and aging (Riera et al., 2014; O’Toole and Jeffery, 2015).

Studies in *C. elegans* have demonstrated that probiotics can improve stress responses and increase the lifespan (Grompone et al., 2012; Martorell et al., 2016; Kumar et al., 2022; Zhang et al., 2022). For example, the administration of *Lactobacillus rhamnosus CNCM I-3690* has been shown to improve survival in *C. elegans* exposed to oxidative stress (Grompone et al., 2012), and the administration of *Lactobacillus plantarum* JBC5 and *Lactobacillus fermentum* strain JDFM216 has been shown to extend the lifespan in *C. elegans* (Park et al., 2018; Kumar et al., 2022).

Importantly, the gut microbiota of *C. elegans* has been shown to play a crucial role in mediating the effects of probiotics on stress responses and aging (Cabreiro and Gems, 2013; Oh et al., 2015; Martorell et al., 2016; Kissoyan et al., 2019; Yang et al., 2019; Dirksen et al., 2020; Poupet et al., 2020; Kumar et al., 2022; Yun et al., 2022). For example, the administration of the probiotic *Bifidobacterium animalis subsp. lactis CECT 8145* reduced fat and mobilized lipids for the metabolism, while modulating the antioxidant response in *C. elegans* (Martorell et al., 2016). However, this effect was dependent on the composition of the differentiated gut microbiota present in the *C. elegans* intestine (Martorell et al., 2016).

The stress responses modulated by probiotic supplementation are a measure of suitability when assessing beneficial probiotic therapy. A few of those measures include the cytoplasmic (cyt), endoplasmic reticulum (ER), and mitochondrial (mt) unfolded protein responses (UPRs), which are important parameters that can be used to evaluate the potential effects of probiotic supplementation in *C. elegans* (Yoneda et al., 2004; Gardner and Walter, 2011; Hetz and Papa, 2018; Kim and Kim, 2018; Martucciello et al., 2020). The UPR_cyt_, UPR_ER_, and UPR_mt_ are cellular stress responses, which are activated during a disruption in protein folding and quality control in the cytoplasm, ER, or mitochondria that can contribute to the stress response in the course of aging and disease (Morley et al., 2002; Cohen and Dillin, 2008; Hipp et al., 2014; Balchin et al., 2016; Maulik et al., 2017; Mamun et al., 2020). However, if probiotic supplementation is found to positively modulate UPR_cyt_, UPR_ER_, and UPR_mt_ activity, it suggests that probiotics can promote improved cytoplasmic, ER, or mitochondrial health or the ability to handle a stress event (Kim and Kim, 2018; Kumar et al., 2022). This, in turn, could have implications for human health, as cytoplasm, ER, or mitochondrial protein quality control is important for maintaining cellular function and preventing the accumulation of misfolded proteins that can lead to diseases such as Parkinson’s disease, cystic fibrosis, or Alzheimer’s disease (Morley et al., 2002; Cohen and Dillin, 2008; Hipp et al., 2014; Balchin et al., 2016; Mamun et al., 2020).

Studies have also shown that probiotics can modulate UPR_cyt_, UPR_ER_, and UPR_mt_ in *C. elegans*. One example of probiotic regulation in *C. elegans* uses the UPR_cyt_ marker, *hsp-16.2*, after supplementation with *Lacticaseibacillus rhamnosus Probio-M9*, which observed no increase in *hsp-16.2* expression (Zhang et al., 2022). This suggests that probiotic supplementation modulates the observed lifespan extension through an hsp-16.2 independent mechanism. The UPR_ER_ was also not modulated by *Lacticaseibacillus rhamnosus Probio-M9*, shown by the normal expression of *hsp-4*, a *C. elegans* marker of ER UPR stress (Zhang et al., 2022; Zhang et al., 2022). However, *Lacticaseibacillus rhamnosus Probio-M9* modulates *hsp-6*, a *C. elegans* marker of UPR_mt_, consistent with the induction of UPR_mt_ stress (Zhang et al., 2022). Studies extending outside of *Lacticaseibacillus rhamnosus Probio-M9* into potential probiotic strains such as *Lacticaseibacillus rhamnosus strain GG*, *Lactobacillus rhamnosus CNCM I-3690*, or *Lactobacillus plantarum* JBC5 come to alternative opinions on whether the mechanistic benefits are positive or negative for lifespan extension, promotion of mitochondrial health, and stress response with probiotic colonization with the induction of a stress response (Grompone et al., 2012; Kumar et al., 2022; Yun et al., 2022; Zhang et al., 2022). The gain or loss of a stress response cannot be assessed in isolation. The stress event needs to be considered along with other endpoint factors such as lifespan extension to assess a positive or negative mechanistic outcome associated with probiotic supplementation.

As such, other important parameters will be used to gage the potential effects of probiotic supplementation in *C. elegans* such as survival, fecundity, and development. *C. elegans*’ short lifespan enables efficient study of interventions affecting survival (Brenner, 1974; Grompone et al., 2012; Martorell et al., 2016; Zhou et al., 2021; Zhou et al., 2022; Kumar et al., 2022; Liu et al., 2022; Yun et al., 2022; Zhang et al., 2022). If probiotic supplementation is found to increase the survival of *C. elegans*, it suggests that probiotics promote better health and longevity. There are many studies that suggest that probiotic supplementation can have beneficial effects on the survival of *C. elegans* under various stress conditions (heat, oxidative, etc.) and may increase the lifespan under normal conditions as well (Grompone et al., 2012; Oh et al., 2015; Martorell et al., 2016; Zhou et al., 2021; Zhou et al., 2022; Kumar et al., 2022; Yun et al., 2022; Zhang et al., 2022). Changes in fecundity can be indicative of alterations in the host’s overall health, such as oxidative stress, inflammation, or altered metabolism (Kumar et al., 2022; Zhang et al., 2022). However, it is important to mention that the effects of probiotics on survival, fecundity, and development may depend on the specific probiotic strains used, the timing and duration of supplementation, and other experimental factors, such as the ability to survive and colonize the gut (Grompone et al., 2012; Kumar et al., 2022; Yun et al., 2022; Zhang et al., 2022).

We investigate the probiotic potential of *L. lactis* and *L. mesenteroides* supplementation on an unhealthy microbiome, simulated by the typical axenic *C. elegans* diet, *E. coli* (OP50) (Brenner, 1974; Cabreiro and Gems, 2013). Our efforts to assess oxidative, UPR_cyt_, UPR_ER_, and UPR_mt_ stress were achieved by observing *gcs-1*, *hsp-16.2*, *hsp-4*, and *hsp-6* stress reporters, respectively, combined with classic health span measurements of survival, fecundity, and development for a wide range of organismal observations of the newly differentiated microbiome (Cabreiro and Gems, 2013; Detienne et al., 2016; Manjarrez and Mailler, 2020; Zhou et al., 2021; Annapure and Nair, 2022; Kumar et al., 2022; Yun et al., 2022; Zhang et al., 2022). The influence of the microbiome on health span is a growing area of interest, with the recent chemotherapeutic advances with fecal transplants efficiently conferring sensitivity to known treatments (Davar et al., 2021). *C. elegans* lacks many of the complex physiological systems found in humans; however, this does not limit the potential of *C. elegans* as a model for probiotic therapy, as it provides valuable insights into many fundamental mechanisms underlying the beneficial effects of probiotics on various metabolic and neurodegenerative diseases (Sonnenburg and Bäckhed, 2016; Kim et al., 2017; Grumezescu and Holban, 2018; Mangiola et al., 2018; Requena et al., 2018; Ticinesi et al., 2019; Annapure and Nair, 2022; Czyż, 2022; Ling et al., 2022; Wang and Zheng, 2022; Marotta, 2023).

## 2 Materials and methods

### 2.1 Culturing *Caenorhabditis elegans*

The *C. elegans* strains used in this study are listed in Supplementary Table S1. Worms were cultured at 20°C on a nematode growth medium (NGM) agar (Brenner, 1974). Plates were seeded with pre-cultured bacterial strains according to the probiotic supplementation method. *C. elegans* were age-synchronized using the egg laying technique and incubated at 20°C until the larvae reached the desired stage of development for subsequent experimentation.

### 2.2 Probiotic supplementation

The bacterial strains used in this study are listed in Supplementary Table S2. A solution of the probiotic supplement *Lactococcus lactis* and *Leuconostoc mesenteroides* was prepared in liquid NGM buffer. The culture was grown overnight at 35°C, concentrated, and resuspended at 15.24 mg/mL. The probiotic solution was added to OP50 at 10% (w/v) and seeded on NGM agar plates using a final concentration of 8 mg/mL. CeMbio cultures were prepared according to the previously designed methods (Dirksen et al., 2020) and seeded on NGM agar plates according to the protocol mentioned previously.

### 2.3 Survival analysis

All survival analysis were performed at 20°C. The L4 stage worms were transferred to fresh plates and used on day 3 for the survival assay (Amrit et al., 2014). The worms were transferred every day until they ceased producing progeny, after approximately 3–5 days and then every 2 days until all worms died, unless indicated otherwise (the plates were spotted for use every 2 days from fresh cultures). For each experiment, at least three plates (25 worms per plate) per bacterial strain were analyzed for the CeMbio survival analysis, and for OP50 supplementation experimentation, five plates (at least 25 worms per plate) per bacterial strain were analyzed. A death event was determined via ceased pharyngeal pumping and no response to gentle prodding with a platinum worm pick. The worms were examined daily. If the worms were unintentionally lost, AVID (age-associated vulval integrity defects frequently described as ruptured) (Leiser et al., 2016), or had undergone matricide, these were censored and excluded from the survival analysis. Statistical analyses were performed using GraphPad Prism 9.5.1 for statistical log-rank (Mantel–Cox) and Gehan–Breslow–Wilcoxon analysis, in all cases *p* <0.05 was considered significant.

### 2.4 Fecundity, body characteristics, and locomotion

Fecundity was measured with five individual L4 synchronized hermaphrodites (five repeats/25 worms in total/bacterial composition). Each individual adult was transferred to fresh plates daily (one worm per plate) until reproduction ceased. The total number of viable offspring was counted per day per worm.

Body characteristics and locomotion were measured from three plates of (at least 20) age-synchronized worms per bacterial strain, at day 1 of adulthood. Videos were taken using a stereo microscope (Nikon S74747) with a D1000 camera and then analyzed using WormLab software (MBF Bioscience). The software analyzed the free roaming locomotion patterns of the worms with the speed metric being reported for this study. These assays were established according to previous recorded methods (Amrit et al., 2014; Keith et al., 2014; Mack et al., 2018; Dirksen et al., 2020).

### 2.5 Intestinal permeability assay

The animals were raised as described previously for lifespan assays. On day 8, the animals were removed from the NGM plates and suspended for 3 h in liquid cultures with blue food dye (FD&C Blue #1, B0790, TCI, 5.0% wt/vol in liquid NGM). The animals were then washed with M9 to remove the unabsorbed dye. Then, the images were captured using a stereo microscope (Nikon S74747) with a D1000 camera for the presence or absence of blue food dye in the body cavity and analyzed using LAS X software (Leica). The following calculation was used to determine the percent of intestinal leakage “permeability”:

$$Smurf\;assay:\%=\frac{\left(\left(intestine+leakage\right)+lenght\;of\;leakage\right)\;\mu m}{\left(body\;cavity+lenght\right)\;\mu m}.$$

Three or more independent experiments were carried out, equaling 8–10 animals per condition. This is as was adapted from the previous methods (Gelino et al., 2016; Kim and Moon, 2019). Data were analyzed using GraphPad Prism version 9.5.1 (GraphPad Software, San Diego, California, United States).

### 2.6 Analysis of stress reporters

The expression of the stress reporters was measured according to Manjarrez and Mailler, 2020, with supporting evidence for heat shock induction of these stress reporters from An and Blackwell (2003), Bar-Ziv et al. (2020), Bischof et al. (2008), Chen et al. (2023), Labbadia et al. (2017), Taylor et al. (2021), Yoneda et al. (2004). The hsp-4::GFP positive control was treated with tunicamycin for 6 h at 20°C, with a 24-h recovery at 20°C prior to imaging (Yoneda et al., 2004; Bischof et al., 2008). As a *hsp-6*p::GFP positive control, 1-day-old worms were heat-shocked for 6 h at 30°C, with a 2-h recovery period at 20°C. All experimental measurements were taken under basal conditions: tunicamycin with 50 ng/mL, or heat-shocked at 35°C, for 30 min followed by a 1-h recovery period at 20°C prior to imaging. The images were acquired using a Leica DMi8 fitted with a SpectraX illuminator (Lumencor), an ORCA Flash4.0 v2 sCMOS camera (Hamamatsu), and LAS X software (Leica). Relative fluorescence units (RFUs) were calculated using a LAS X relative fluorescence calculator using a 200 × 200-µm square as a background measurement for the fluorescence intensity of the worm. F(t) = fluorescence channel/region of interest (ROI); F(0) = fluorescence channel/background (Bkg), and K is set to 1 as normalized EGFP (Stepanenko et al., 2008):

$$F=\frac{dF}{F\left(0\right)}=K*\frac{F\left(t\right)}{F\left(0\right)}\;\frac{F\left(0\right)}{Fb}.$$

Upregulation of the positive control for each stress reporter was used to obtain the F_max_ (maximum reporter intensity) (Manjarrez et al., 2020; Manjarrez and Mailler, 2020). The normalized values were plotted, and *p*-values were generated by the nested *t*-test using GraphPad Prism version 9.5.1 (GraphPad Software, San Diego, California, United States).

### 2.7 Statistics and reproducibility

Prism 9.5.1 software was used for the survival analysis, using the log-rank (Mantel–Cox) method which analyzed the significance of difference in the overall curve. The Gehan–Breslow–Wilcoxon method was used to assess the significance of survival earlier versus later in the survival timeline. The statistical analysis resulting from the Mantel–Cox, Gehan–Breslow–Wilcoxon, and nested and Student’s *t*-test, in all cases, showed that *p* <0.05 was considered significant. An asterisk, in the figures, indicates statistical significance of the aforementioned statistical analysis as compared to its indicated reference. At least three biological replicates comprise all the referenced datasets.

## 3 Results

### 3.1 Survival

**FIGURE 1:**
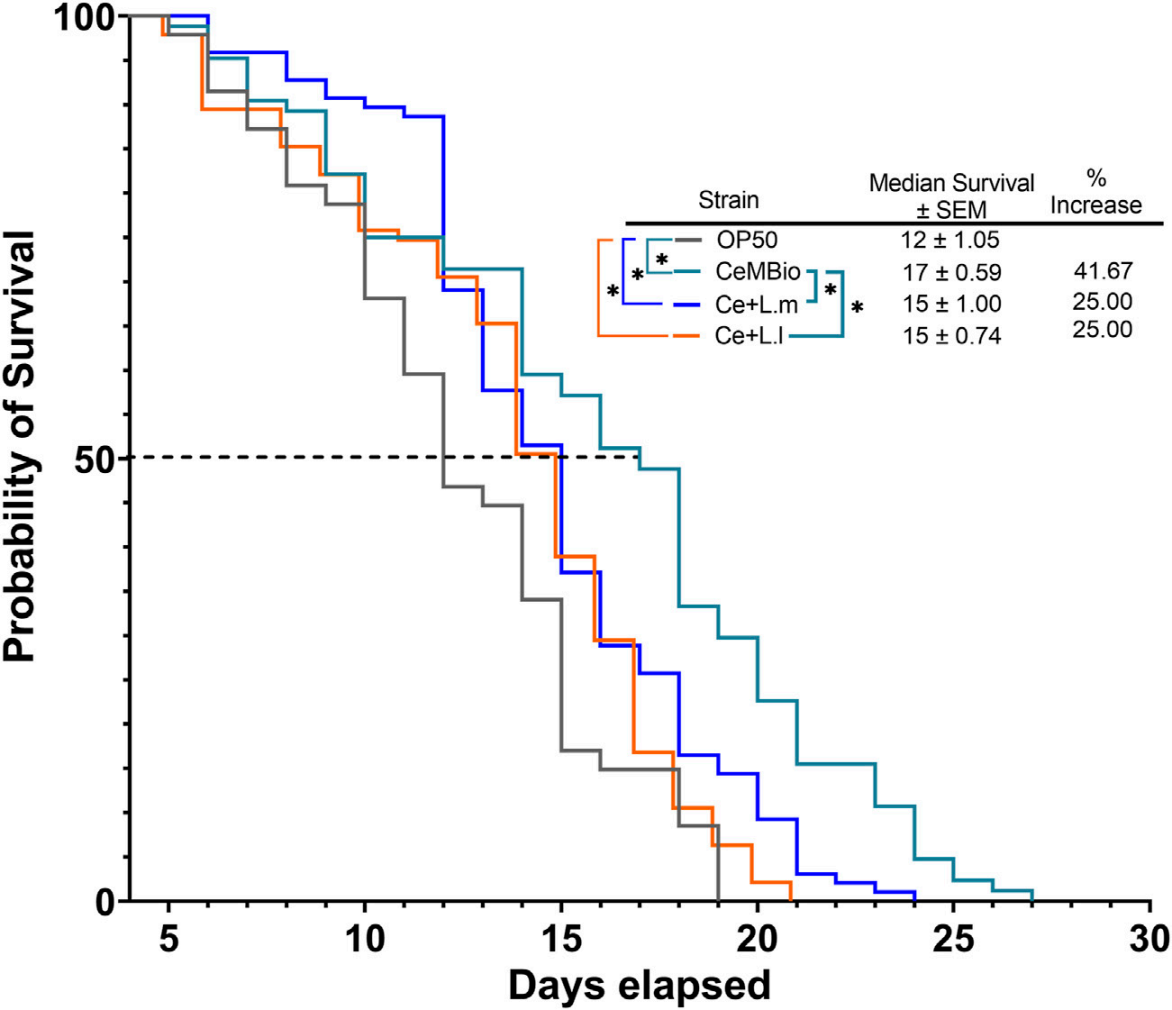
Effects of *L. lactis* and *L. mesenteroides* combined with CeMbio feeding on the regulation of survival in *C. elegans*, *L. lactis,* and *L. mesenteroides*, resuspended in CeMbio, were tested for the lifespan extension of wild-type N2 worms (*p* <0.05, log-rank test). Survival assays were determined in at least three independent experiments (OP50, dark gray line; CeMbio, teal line; CeMbio + *L. mesenteroides* (Ce + L.m), blue line; and CeMbio + *L. lactis* (Ce + L.l), orange line).

The effect of CeMbio, the laboratory-derived microbiome based on natural isolates, and CeMbio supplemented with *L. lactis* or *L. mesenteroides* on the survival of *C. elegans* was compared to that of the commonly used *E. coli*, OP50. The results showed that all three CeMbio treatments exhibited significant differences in survival compared to OP50 (Figure 1). While CeMbio showed the longest median survival and overall lifespan when supplemented with *L. lactis* or *L. mesenteroides*, it demonstrated a reduction in the median survival and overall lifespan, contrary to our initial expectation (Figure 1). This survival analysis suggests that the supplementation of *L. lactis* or *L. mesenteroides* to CeMbio had a negative effect on the balance of the differentiated CeMbio microbial community.

**FIGURE 2:**
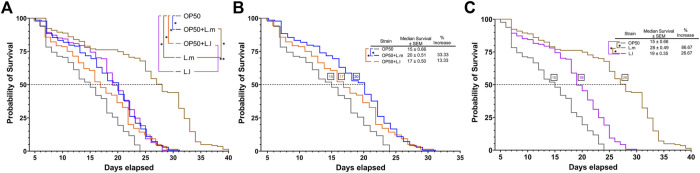
Effects of *L. lactis* and *L. mesenteroides* and combined with OP50 feeding on the regulation of survival in *C. elegans* ***.* (A)** *L. lactis* and *L. mesenteroides* along with resuspended *L. lactis* and *L. mesenteroides* in OP50 tested for the lifespan extension of wild-type N2 worms (*p* <0.05, log-rank test) or the Gehan–Breslow–Wilcoxon test (**, *p* <0.05). **(B)** Resuspended *L. lactis* and *L. mesenteroides* in the OP50 lifespan extension of wild-type N2 worms (*, *p* <0.05, log-rank test) or the Gehan–Breslow–Wilcoxon test (**, *p* <0.05). **(C)** Monocultures of *L. lactis* and *L. mesenteroides* tested for the lifespan extension of wild-type N2 worms (*p* <0.05, log-rank test). Survival assays were determined in at least three independent experiments (OP50, dark gray line; OP50 + *L. mesenteroides* (OP50 + L.m), blue line; OP50 + *L. lactis* (OP50 + L.l), orange line; *L. mesenteroides* (L.m), brown line; and *L. lactis* (L.l), purple line).

This led to the possibility that supplementation of either *L. lactis* or *L. mesenteroides* to the undifferentiated OP50 laboratory strain would improve the lifespan and median survival of the nematodes compared to the OP50 alone, in which both lactic acid bacteria strains and combined OP50 conditions are shown to colonize the *C. elegans* gut (S1). After investigating the effect of supplementing OP50 with *L. lactis* or *L. mesenteroides*, a positive correlation was discovered with the extension of the median and overall lifespan, without showing any signs of developmental arrest associated with either potential probiotic strain (Figures 2A–C; Supplementary Figure S2). These results suggest that the nutrients/metabolites derived by supplementing *L. lactis* or *L. mesenteroides* with OP50 must have advantageous effects by differentiating the *C. elegans* axenic OP50 strain. Most of the lactic acid bacterial strains or supplementations exhibited extensions in the median and overall lifespan within 13.33%–33.33% and 25%–29%, respectively. The *L. lactis-*supplemented OP50 or *L. lactis* monoculture only shows significant differences when analyzed for early death events by the Gehan–Breslow–Wilcoxon test. The log-rank test proved insignificant between the supplementation and the monoculture for *L. lactis*. However, additional support for the beneficial contribution of nutrients/metabolites of *L. mesenteroides* intensified with the growth on the monoculture, which exhibits a lifespan extension that exceeds of all biomes tested (Figure 2C), with an 87% increase in the median survival and a 67% increase in the overall lifespan beyond the standard OP50. While *L. mesenteroides* is not known to produce antimicrobials such as nisin, *L lactis* has been reported to produce nisin (Khelissa et al., 2021). A significant reduction in the survival rate of *C. elegans* has been observed with exposure to nisin concentrations higher than 0.2 mg mL^−1^ (Boelter et al., 2023). However, since the addition of lactic acid bacteria to OP50 has led to a significant improvement in the median and overall survival in simulated biomes, the deleterious effect of nisin produced (if any) by *L. lactis* was not observed.

### 3.2 Morphology and locomotive behavior

**FIGURE 3:**
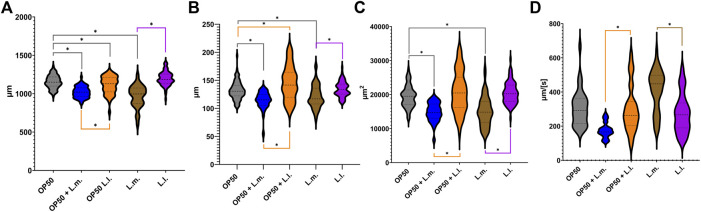
Effects of *L. lactis* and *L. mesenteroides* and combined with OP50 feeding on the body characteristics of *C. elegans*. Cumulative groups of the five combinations were analyzed in relation to the control, OP50, and its related supplementation partner and strain. The color of the bracket indicates the higher significant value of the nested *t*-test (*, *p* <0.05). **(A)** Length of N2 *C. elegans* grown on the supplemented bacterial strains. **(B)** Width of N2 *C. elegans* grown on the supplemented bacterial strains. **(C)** Area of the N2 *C. elegans* grown on the supplemented bacterial strains. **(D)** Speed of the N2 *C. elegans* grown on the supplemented bacterial strains. Body characteristic assays were determined in at least three independent experiments (OP50, dark gray; OP50 + *L. mesenteroides* (OP50 + L.m), blue; OP50 + *L. lactis* (OP50 + L.l), orange; *L. mesenteroides* (L.m), brown; and *L. lactis* (L.l), purple).

In the OP50 + L.m. group, the nematodes were found to be morphologically distinct, being shorter, thinner, and possessing a smaller area than their counterparts in the OP50 and OP50 + L.l. groups (Figures 3A–C; Supplementary Figure S3). Additionally, these nematodes displayed slower locomotor behavior compared to those in the OP50, OP50 + L.l., and L.m. groups (Figure 3D; Supplementary Figure S3). Nematodes in the OP50 + L.l. group were shorter and wider than those in the OP50 group, yet longer and wider than those in the OP50 + L.m. group (Figures 3A, B; Supplementary Figure S3). They were significantly larger in area and displayed faster locomotion than those in the OP50 + L.m. group, but did not significantly differ from the OP50 group in these aspects (Figures 3C, D; Supplementary Figure S3). The L.m. group nematodes were shorter, thinner, and smaller than their counterparts in the OP50, OP50 + L.l., and L.l. groups. However, they displayed faster locomotive behavior than the OP50 + L.m., OP50 + L.l., and L.l groups (Figures 3A–D; Supplementary Figure S3). In the L.l. group, nematodes were longer, wider, and larger in area than their L.m. counterparts (Figures 3A–C; Supplementary Figure S3). Interestingly, two distinct widths were observed in this group, with measurements varying around the mean (Figure 3B). These nematodes exhibited slower locomotive behaviors than those in the L.m. group (Figure 3D).

### 3.3 Progeny production

**FIGURE 4:**
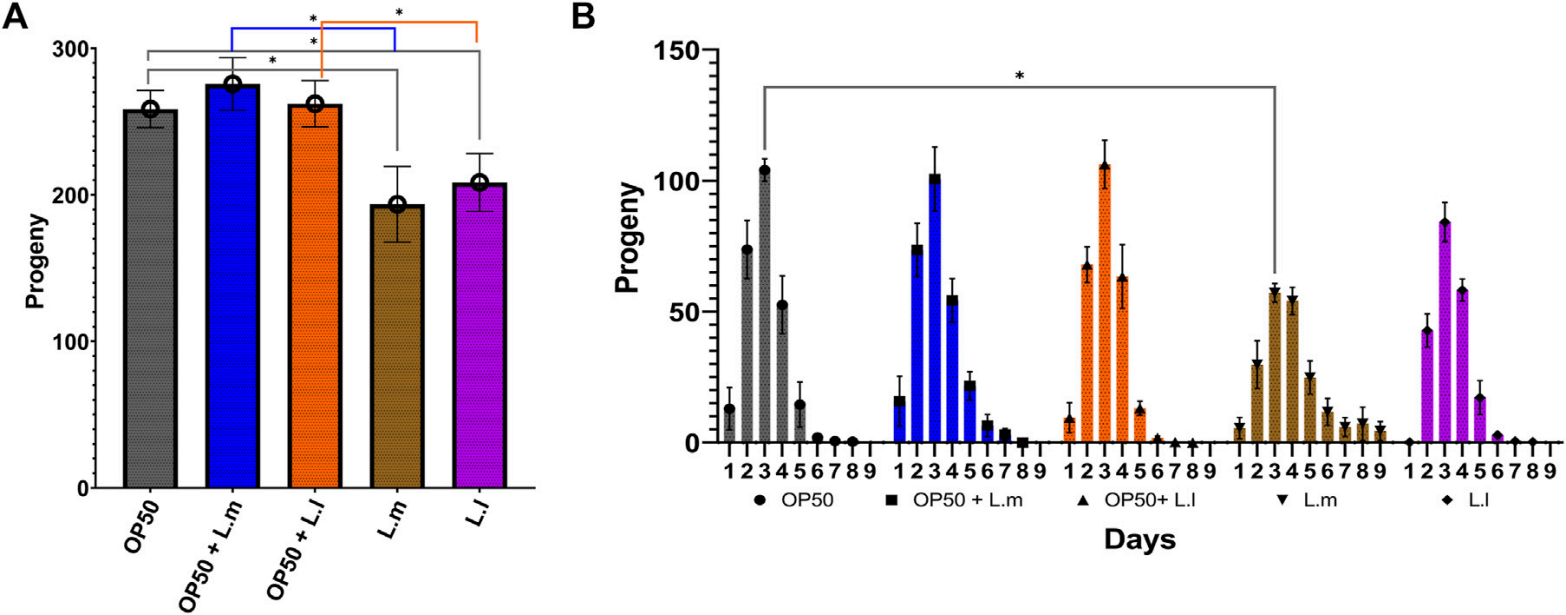
Effects of *L. lactis* and *L. mesenteroides* and combined with OP50 feeding on the fecundity of *C. elegans*. Cumulative groups of the five combinations were analyzed in relation to the control, OP50, and its related supplementation partner and strain. The color of the bracket indicates the higher significant value of the nested *t*-test (*, *p* <0.05). **(A)** Average number of total progenies. **(B)** Average number of progenies per day. Fecundity assays were determined in at least three independent experiments (OP50, dark gray; OP50 + *L. mesenteroides* (OP50 + L.m), blue; OP50 + *L. lactis* (OP50 + L.l), orange; *L. mesenteroides* (L.m), brown; and *L. lactis* (L.l), purple).

In terms of progeny production, the OP50 + L.m. group, despite their reduced speed, produced a higher number of progeny than the L.m. monoculture (Figure 4A). The OP50 + L.l. group produced progeny equivalent to those of the OP50 group and at a higher level than those of the L.l. monoculture (Figure 4A). The L.l. group produced fewer progeny than both the OP50 and OP50 + L.l. groups (Figures 4A, B). The L.m. group showed a decrease in progeny production on the third day of the reproductive cycle compared to the OP50 group and produced fewer total progeny than the OP50 and OP50 + L.m. groups (Figures 4A, B). Despite this, the L.m. group continued to produce progeny for a longer duration at a higher level than those of the other groups (Figure 4B).

### 3.4 Intestinal permeability

**FIGURE 5:**
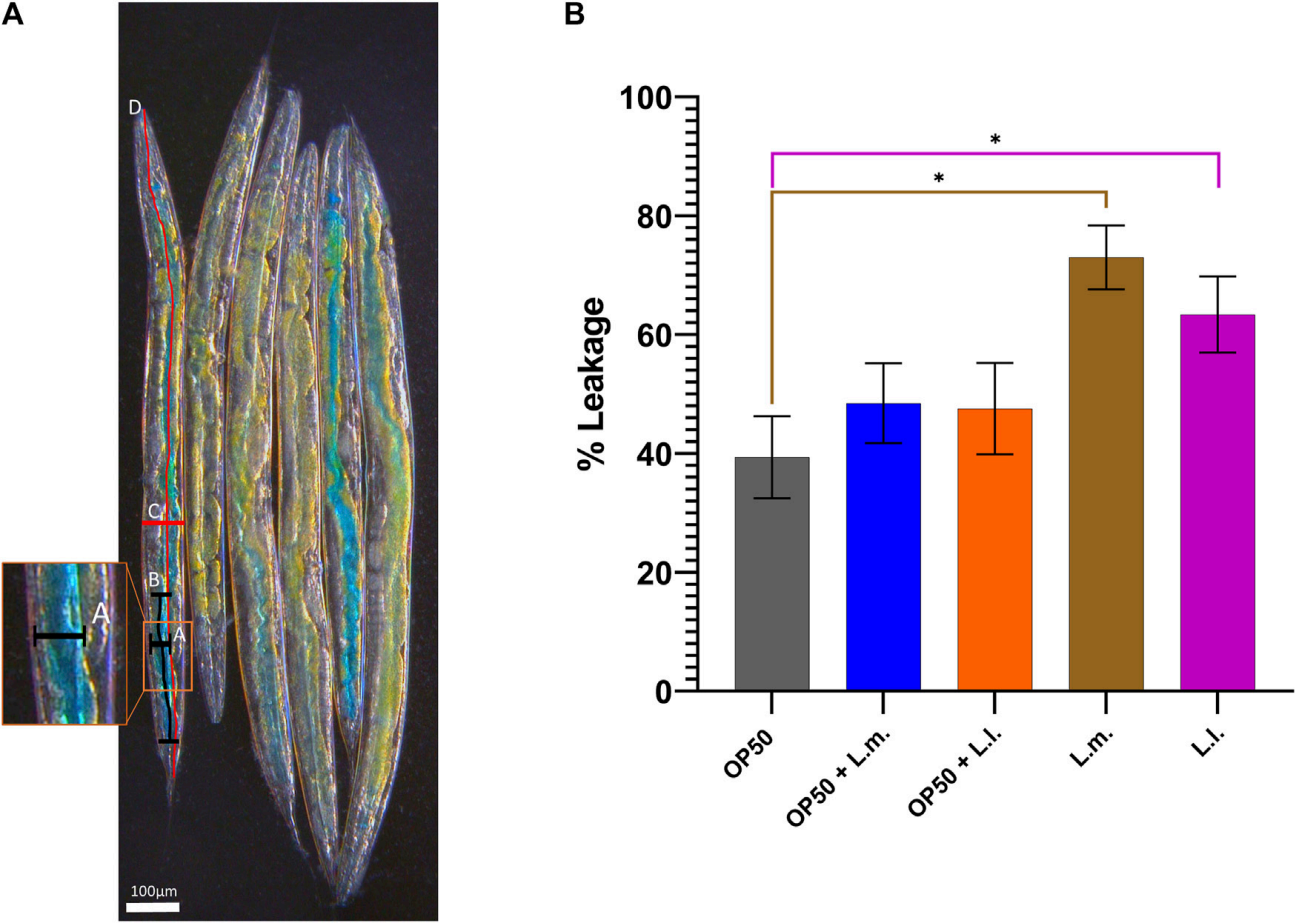
Effects of *L. lactis* and *L. mesenteroides* and combined with OP50 on the intestinal permeability of wild-type *C. elegans*. Cumulative groups of the five combinations were analyzed in relation to the control, OP50, and its related supplementation partner and strain. The color of the bracket indicates the higher significant value of the nested *t*-test (*, *p* <0.05). **(A)** Control OP50 worms as a visual representation of the Smurf assay. It is detailed with a diagram of the measurement according to the formula $\%=\frac{\left(\left(intestine+leakage\right)+lenght\;of\;leakage\right)\;\mu m}{\left(body\;cavity+lenght\right)\;\mu m}$ , whereas $\%=\frac{\left(\left(A\right)+B\right)\;\mu m}{\left(C+D\right)\;\mu m}$ . **(B)** Percentage of the intestinal permeability-based leakage of the blue dye into the body cavity. Smurf assays were determined in at least three independent experiments (OP50, dark gray; OP50 + *L. mesenteroides* (OP50 + L.m), blue; OP50 + *L. lactis* (OP50 + L.l), orange; *L. mesenteroides* (L.m), brown; and *L. lactis* (L.l), purple).

Assessing intestinal permeability using the Smurf assay revealed an increase in 8-day-old L.m. worms compared to the OP50 group (Figure 5). Similarly, an increased intestinal permeability was observed in the 8-day-old L.l. group, indicating that these longer-lived worms also had increased intestinal permeability akin to the L.m. monoculture group (Figure 5). However, there was no significant increase in the intestinal permeability that was observed in the OP50 + L.m. or OP50 + L.m. group (Figure 5).

### 3.5 Reactive oxygen species stress response

**FIGURE 6:**
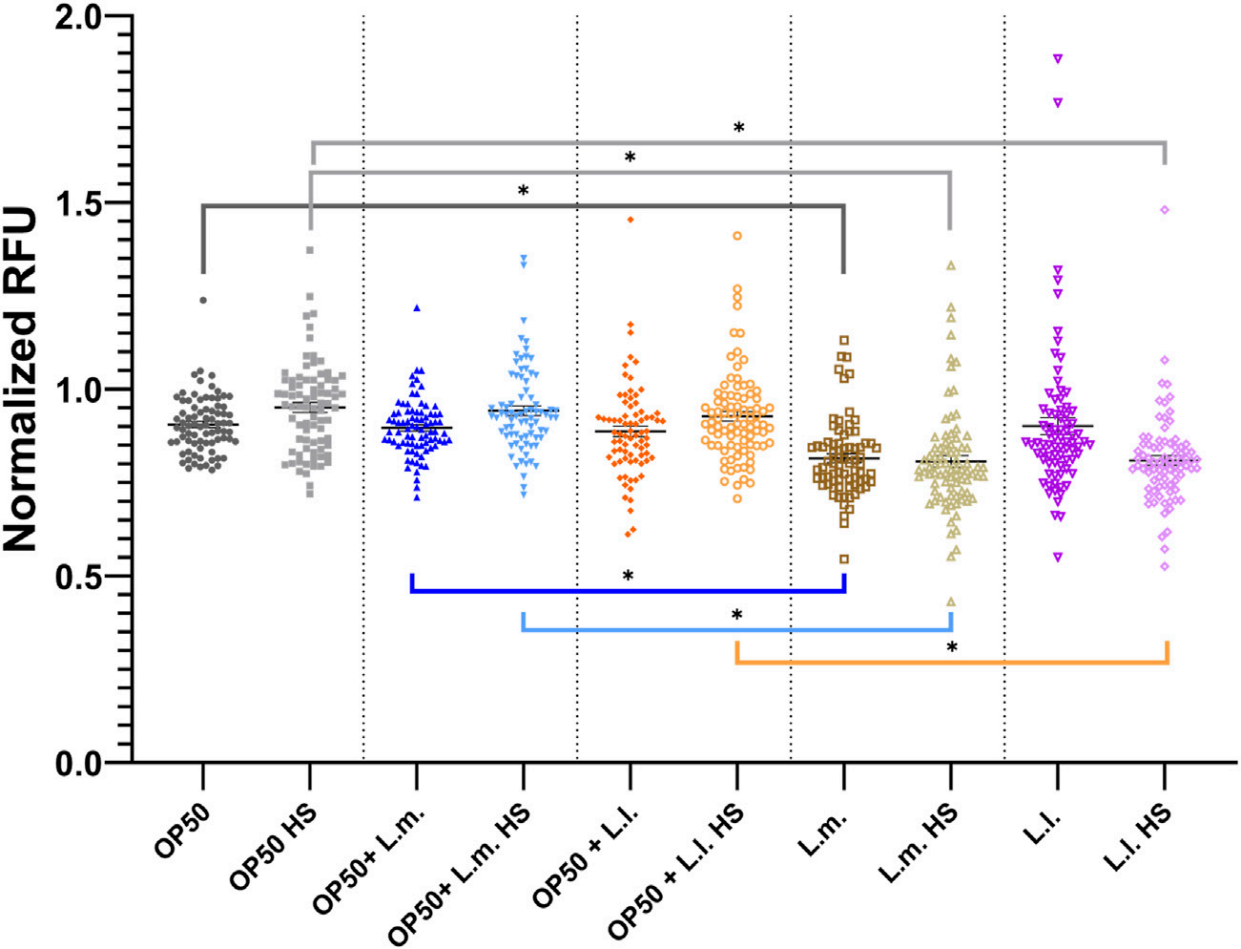
Effects of *L. lactis* and *L. mesenteroides* and combined with OP50 on the reactive oxygen species reporter *gcs-1p::GFP*. Cumulative groups of the five combinations were analyzed in relation to the control, OP50, and its related supplementation partner and strain for both the basal and heat-shocked group. The color of the bracket indicates the higher significant value of the nested *t*-test (*, *p* <0.05). Stress assays were determined in at least three independent experiments (OP50, dark gray; OP50 HS, light gray; OP50 + *L. mesenteroides* (OP50 + L.m), blue; OP50 + L.m. HS, light blue; OP50 + *L. lactis* (OP50 + L.l), orange; OP50 + L.l. HS, light orange; *L. mesenteroides* (L.m), brown; L.m., light brown; *L. lactis* (L.l), purple; L.l. HS, light purple).

In the context of reactive oxygen species (ROS) stress responses, the data showed that the basal and heat shock (HS) levels in the OP50 + L.m. group were elevated compared to those in the L.m. group (Figure 6; Supplementary Figure S4). Despite this increase, the basal and HS levels remained relatively unchanged upon extrinsic heat shock insults. In the OP50 + L.l. HS group, the ROS stress response was found to be elevated compared to that in the L.l. HS group. However, similar to the OP50 + L.m. group, the basal and HS levels remained relatively unchanged upon insults (Figure 6; Supplementary Figure S4). In the L.m. group, the basal ROS levels were found to be below those in the OP50 and OP50 + L.m. group, as well as the HS groups for these culture groups (Figure 6; Supplementary Figure S4). The basal and HS ROS response levels in the L.m. group remained relatively unchanged upon stress insults as measured by the *gcs-1* reporter strain, indicating the lowest measured stress levels (Figure 6; Supplementary Figure S4). The ROS stress response in the L.l. HS group was found to be below that of the OP50 HS and OP50 + L.l. HS group (Figure 6; Supplementary Figure S4). However, the basal group showed a slight increase over the HS group but was otherwise unchanged upon insult (Figure 6; Supplementary Figure S4).

### 3.6 Unfolded protein response (UPR_cyt_) stress response

**FIGURE 7:**
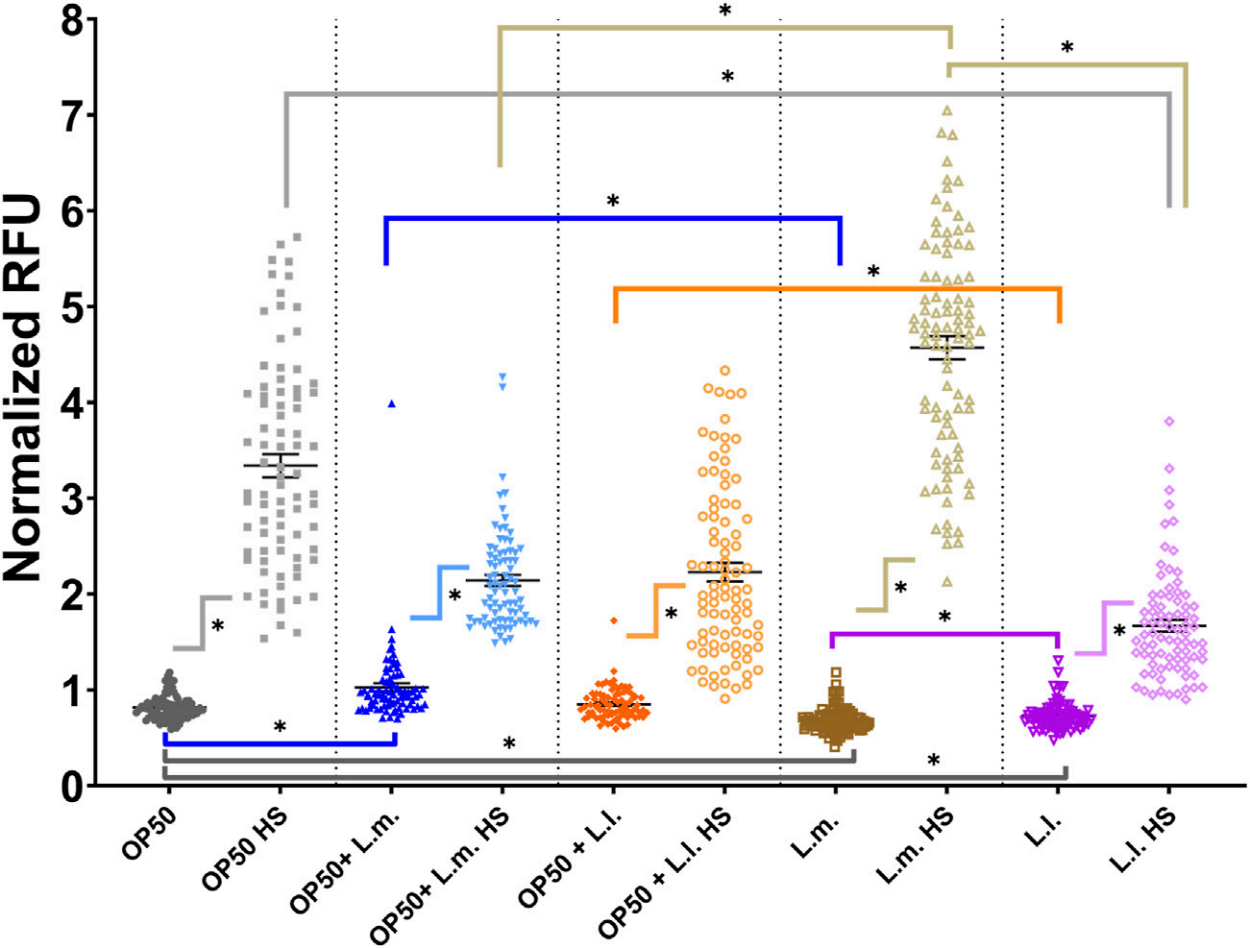
Effects of *L. lactis* and *L. mesenteroides* and combined with OP50 on the cytoplasmic unfolded protein response reporter *hsp-16.2p::GFP*. Cumulative groups of the five combinations were analyzed in relation to the control, OP50, and its related supplementation partner and strain for both the basal and heat-shocked group. The color of the bracket indicates the higher significant value of the nested *t*-test (*, *p* <0.05). Stress assays were determined in at least three independent experiments (OP50, dark gray; OP50 HS, light gray; OP50 + *L. mesenteroides* (OP50 + L.m), blue; OP50 + L.m. HS, light blue; OP50 + *L. lactis* (OP50 + L.l), orange; OP50 + L.l. HS, light orange; *L. mesenteroides* (L.m), brown; L.m., light brown; *L. lactis* (L.l), purple; L.l. HS, light purple).

Concerning the UPR_cyt_ stress responses, interesting patterns were observed across different groups. In the OP50 + L.m. group, the basal stress response levels were significantly higher than those in the OP50 and L.m. group. However, these levels were decreased in comparison to the OP50 + L.m. HS and L.m. HS response (Figure 7; Supplementary Figure S5). For the OP50 + L.l. group, the basal stress response levels were notably decreased compared to both the OP50 + L.l. HS and L.l. groups (Figure 7; Supplementary Figure S5). Thus, the OP50 + L.l. group displayed a reduced UPR_cyt_ basal stress response. In the L.m. group, the UPR_cyt_ basal stress response was significantly decreased compared to the OP50, OP50 + L.m., and L.l. group (Figure 7; Supplementary Figure S5). However, the L.m. HS stress response in L.m. showed a robust increase over basal L.m. levels, with OP50 + L.m. HS, and L.l. HS, suggesting an elevated UPR_cyt_ stress response upon heat shock in the L.m. group. Lastly, in the L.l. group, the UPR_cyt_ basal level was decreased relative to both the L.l. HS, OP50, and OP50 + L.l. groups (Figure 7; Supplementary Figure S5). The L.l. HS stress response decreased compared to the OP50 HS and L.m. HS stress responses, indicating a lowered UPR_cyt_ response upon heat shock in the L.l. group.

### 3.7 Unfolded protein response (UPR_ER_) stress response

**FIGURE 8:**
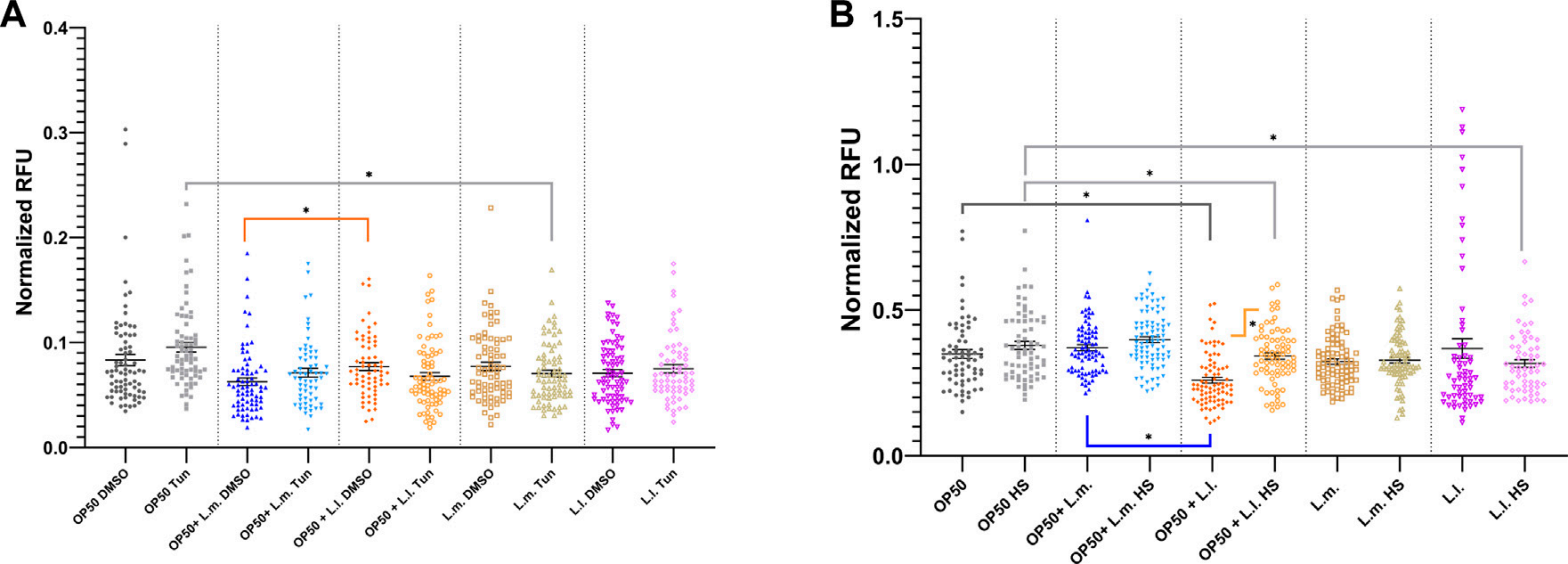
Effects of *L. lactis* and *L. mesenteroides* and combined with OP50 on the endoplasmic reticulum unfolded protein response reporter *hsp-4::GFP*. **(A)** Cumulative groups of the five combinations were analyzed in relation to the control, OP50, and its related supplementation partner and strain for both the basal and tunicamycin-treated group. **(B)** Cumulative groups of the five combinations were analyzed in relation to the control, OP50, and its related supplementation partner and strain for both the basal and heat-shocked group. The color of the bracket indicates the higher significant value of the nested *t*-test (*, *p* <0.05). Stress assays were determined in at least three independent experiments (OP50, dark gray; OP50 HS, light gray; OP50 + *L. mesenteroides* (OP50 + L.m), blue; OP50 + L.m. HS, light blue; OP50 + *L. lactis* (OP50 + L.l), orange; OP50 + L.l. HS, light orange; *L. mesenteroides* (L.m), brown; L.m., light brown; *L. lactis* (L.l), purple; and L.l. HS, light purple).

In terms of UPR_ER_ stress responses, short-term treatment with tunicamycin showed only marginal increases although not significant for OP50, OP50 + L.m., and L.l. While, showing decreases with short-term exposure for OP50 + L.l and L.m. compared to controls (Figure 8A; Supplementary Figure S6A). OP50 + L.l did show an increase amount of basal stress over OP50 + L.m under DMSO treatment but did not show significant differences upon tunicamycin treatment (Figure 8A). However, the OP50 tunicamycin treated group showed a significant increase in stress over the L.m. treated group upon short-term exposure (Figure 8A; Supplementary Figure S6A). The other possible induction of the hsp-4 transgene according to the CGC, heat shock, shows a diverse trend across the different groups studied. For the OP50 + L.m. group, the UPR_ER_ basal stress response levels were found to be elevated above those in the OP50 + L.l. group. A slight elevation in the HS to basal level was observed, although the changes in these levels upon extrinsic heat shock insults remained relatively unchanged (Figure 8B; Supplementary Figure S6B). In the OP50 + L.l. group, the levels in the UPR_ER_ HS group were significantly elevated compared to the basal response levels in the same group. These levels were also found to be decreased relative to the OP50 and OP50 HS groups. Furthermore, the OP50 + L.l. response level decreased compared to the OP50 + L.m. response level (Figure 8B; Supplementary Figure S6B). In the L.m. group, the UPR_ER_ basal and L.m. HS stress responses remained relatively unchanged upon insult, suggesting that this group had robust resistance to UPR_ER_ stress (Figure 8B; Supplementary Figure S6B). No significant differences were observed between these responses and those of the other groups. In the L.l. group, the UPR_ER_ HS group was found to decrease relative to the OP50 HS group, suggesting a reduced response upon heat shock in the L.l. group. Interestingly, the basal groups showed a slight increase over the HS groups or slight increase or decreases in most groups with tunicamycin treatment, however; these changes in most treatments were not significant, and the responses were otherwise relatively unchanged (Figures 8A, B; Supplementary Figures S6A, B).

### 3.8 Unfolded protein response (UPR_mt_) stress response

**FIGURE 9:**
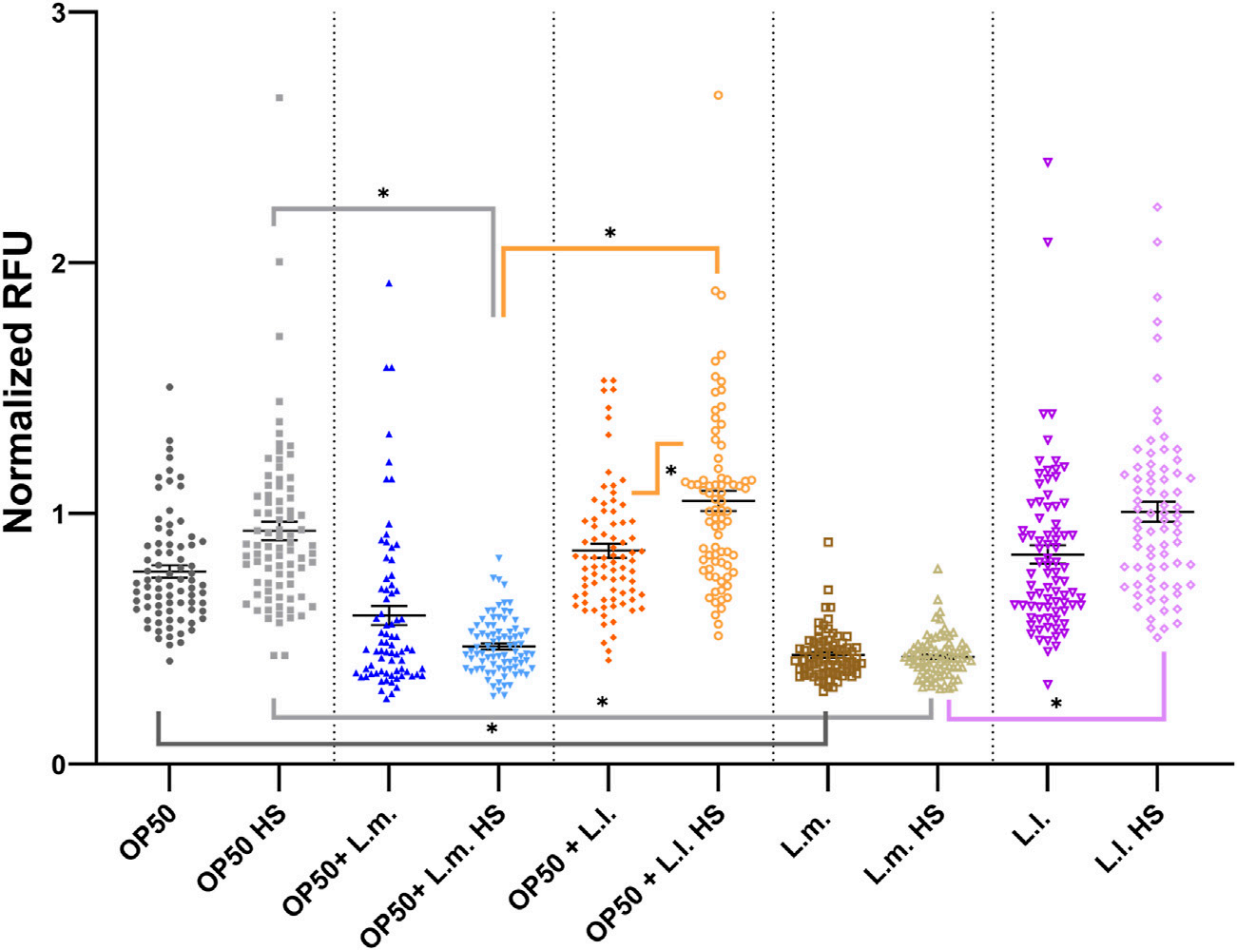
Effects of *L. lactis* and *L. mesenteroides* and combined with OP50 on the endoplasmic reticulum unfolded protein response reporter *hsp-6p::GFP*. Cumulative groups of the five combinations were analyzed in relation to the control, OP50, and its related supplementation partner and strain for both the basal and heat-shocked group. The color of the bracket indicates the higher significant value of the nested *t*-test (*, *p* <0.05). Stress assays were determined in at least three independent experiments (OP50, dark gray; OP50 HS, light gray; OP50 + *L. mesenteroides* (OP50 + L.m), blue; OP50 + L.m. HS, light blue; OP50 + *L. lactis* (OP50 + L.l), orange; OP50 + L.l. HS, light orange; *L. mesenteroides* (L.m), brown; L.m., light brown; *L. lactis* (L.l), purple; L.l. HS, light purple).

The UPR_mt_ stress responses across various groups demonstrated intriguing patterns. In the OP50 + L.m. group, the HS response showed a marked decrease compared to the OP50 HS and OP50 + L.l. HS responses (Figure 9; Supplementary Figure S7). However, under these conditions, the basal level of response was slightly elevated over the OP50 + L.m. HS levels, but this elevation was not significant (Figure 9; Supplementary Figure S7). In the context of the OP50 + L.l. group, the level of the UPR_mt_ HS group was elevated compared to the basal response level (Figure 9; Supplementary Figure S7). While the level of the OP50 + L.l. HS group was increased above the level of the OP50 + L.m. HS group, there were no significant differences between OP50 HS or L.l. HS groups (Figure 9; Supplementary Figure S7). Investigating the L.m. group, the UPR_mt_ showed that the L.m. basal and L.m. HS stress responses were relatively unchanged upon insults, indicating the lowest measured stress levels. Basal and HS stress responses in L.m. were found to be decreased below those in the OP50 and OP50 HS groups (Figure 9; Supplementary Figure S7). Moreover, L.m. HS stress response levels decreased relative to those in the L.l. HS group (Figure 9; Supplementary Figure S7). In the L.l. group, the UPR_mt_ showed a slight increase in the HS response over the basal condition upon insult, but this was not significant (Figure 9; Supplementary Figure S7). However, the L.l. HS group exhibited an increased response compared to the L.m. HS group, while maintaining an overall higher response in the basal and HS level (Figure 9; Supplementary Figure S7).

## 4 Discussion

The use of probiotics, particularly lactic acid bacteria, has been increasingly studied for its potential health benefits. One of the most promising areas of research has been in its impact on the lifespan, stress response, and nutrient uptake. Several studies have shown that probiotics can lead to a longer lifespan in various organisms, including *C. elegans*. In addition to the impact of probiotics on the lifespan, they have also been found to regulate stress responses more tightly. For instance, a study published in PNAS in 2011 found that the probiotic *Lactobacillus reuteri* reduced stress-induced cortisol levels in mice (Bravo et al., 2011). This suggests that the introduction of probiotics can help individuals better manage stress, which is essential for their overall health and wellbeing.

The current study demonstrates that significantly lower basal and stress levels compared to controls are indicative of a positive early health response. These findings were consistent across different cultured groups, which were most significant in the *L*. *mesenteroides* monoculture, emphasizing the potential probiotic applicability of these biomarkers in predicting early health responses in the *C. elegans* model system (Dhama et al., 2019). The relationship between *L*. *mesenteroides*’ stress response and survival probability reveals that a balanced and tightly modulated stress response is associated with the longest survival probability (Vermeulen and Loeschcke, 2007). Upon extrinsic stress insults, *L*. *mesenteroides* showed a capacity to buffer stress with relatively unchanged stress responses and was associated with a significantly higher survival probability compared to those with the most altered basal-to-stress responses, OP50 + L.l. This suggests the accumulation of protective compounds in the *L*. *mesenteroides* group, consistent with the uptake of additional advantageous nutrients from this probiotic supplement (Miyamoto et al., 2023).

The analysis of stress response-related biomarkers revealed that *L*. *mesenteroides*, with a stable stress response, exhibited minimal fluctuations in the levels of the ROS, UPR_ER_, and UPR_mt_ response genes, suggesting an efficient adaptation mechanism to maintain homeostasis (Grompone et al., 2012; Park et al., 2018; Dhama et al., 2019; Kumar et al., 2022; Yun et al., 2022; Zhang et al., 2022). This well-regulated stress response could play a pivotal role in boosting the organism’s resilience and ability to cope with insults, ultimately leading to increased survival probabilities provided through probiotic supplementation with the *L*. *mesenteroides* culture. Although this was seen most prevalently in the *L*. *mesenteroides* monoculture, the supplementation of the OP50 axenic culture with *L*. *mesenteroides*, OP50 + L.m., also showed a similar trend, only not to the same degree (Miyamoto et al., 2023).

Furthermore, the study revealed that *L*. *mesenteroides* with relatively unchanged stress responses exhibited better overall health span parameters, including anatomically beneficial features, which contribute to their longer survival probability and ability to maintain stability during a stress response. However, contradictory to current findings, *L. mesenteroides* and *L*. *lactis* show increased intestinal permeability, even with increased median and overall survival probabilities (Gelino et al., 2016; Kim and Moon, 2019). Even though intestinal permeability has been shown to be associated with irritable bowel syndrome, obesity, chronic kidney disease, and cardiovascular diseases, it shows a link between increased permeability and the promotion of dysbacteriosis (Inczefi et al., 2022). However, another concept is that probiotics can lead to a more permeable intestine, which can lead to more efficient nutrient uptake. This is owing to the fact that probiotics can promote the growth of beneficial bacteria in the gut, which can aid in digestion and nutrient absorption (O’Toole and Jeffery, 2015; Requena et al., 2018; Annapure and Nair, 2022). In turn, this can lead to improved overall health and increased energy levels. As such, the maintenance of a physiological balance in the face of stressors could be a key feature determining the longevity and overall health of an animal provided with probiotic supplementation (Grompone et al., 2012; Oh et al., 2015; Park et al., 2018; Kumar et al., 2022; Yun et al., 2022; Zhang et al., 2022).

In conclusion, the results of this study underscore the importance of a balanced and tightly controlled stress response for ensuring the longest survival probability upon oxidative and proteostatic insult. The ability to maintain homeostasis and efficiently buffer extrinsic stressors appears to be a critical determinant of an organism’s resilience and survival. Moreover, the efficacy of probiotics can vary depending on the individual’s gut microbiome and health status. The use of probiotics has shown promising results for improving the lifespan, stress response, and nutrient uptake. Consequently, the incorporation of probiotics into one’s diet or taking probiotic supplements may provide significant health benefits. Despite the promising findings using these potential probiotics, further research is still needed to fully elucidate the molecular mechanisms underlying this phenomenon and to explore potential therapeutic strategies to enhance stress response regulation and improve survival outcomes through the probiotic application of *L*. *lactis* and *L*. *mesenteroides*.

### 4.1 Summary

This study highlights the importance of significantly lower basal and stress levels as indicators of an early health response in the *C. elegans* model system. The results emphasize the potential probiotic applicability of these biomarkers for predicting early health responses, whereas a balanced and tightly modulated stress response was found to be associated with the longest survival probability, which demonstrated significantly longer survival rates than those with altered stress responses. The efficient adaptation mechanisms that maintain homeostasis ultimately lead to an increased survival probability. The relatively unchanged stress responses exhibited better overall health span parameters, contributing to the ability to maintain physiologically balanced condition in the face of stressors, which is a key feature in determining the longevity and overall health of an organism provided with *L*. *lactis* and *L*. *mesenteroides* as a therapeutic probiotic supplement.

## Data Availability

The raw data supporting the conclusion of this article will be made available by the authors, without undue reservation.
