# Supplementary material for: Probiotic effects of Lactococcus lactis and Leuconostoc mesenteroides on stress and longevity in Caenorhabditis elegans
Source: Front Physiol. 2023 Sep 12;14:1207705. doi: 10.3389/fphys.2023.1207705 (PMC10522913; doi:10.3389/fphys.2023.1207705)
Supplement: Supplementary file 1 [file DataSheet2.PDF]

**Strain Name**	**Source**	**Transgene**	**Assay**
N2 (Bristol Strain)	Ken Miller	none	Survival/Fecundity/Intestinal Permeability/Growth
LD1171	CGC	ldIs3 [gcs-1p::GFP + rol-6(su1006)]	Oxidative Stress
CL2070	CGC	dvIs70 [hsp-16.2p::GFP + rol-6(su1006)]	Stress assay (UPR_{Cyt})
GL347	CGC	zcIs13 [hsp-6p::GFP + lin-15(+)]	Stress assay (UPR_{mt})
SJ4005	CGC	zcIs4 [hsp-4::GFP]	Stress assay (UPR_{ER})
