# Supplementary figures and images for: Probiotic effects of Lactococcus lactis and Leuconostoc mesenteroides on stress and longevity in Caenorhabditis elegans
Source: Front Physiol. 2023 Sep 12;14:1207705. doi: 10.3389/fphys.2023.1207705 (PMC10522913; doi:10.3389/fphys.2023.1207705)

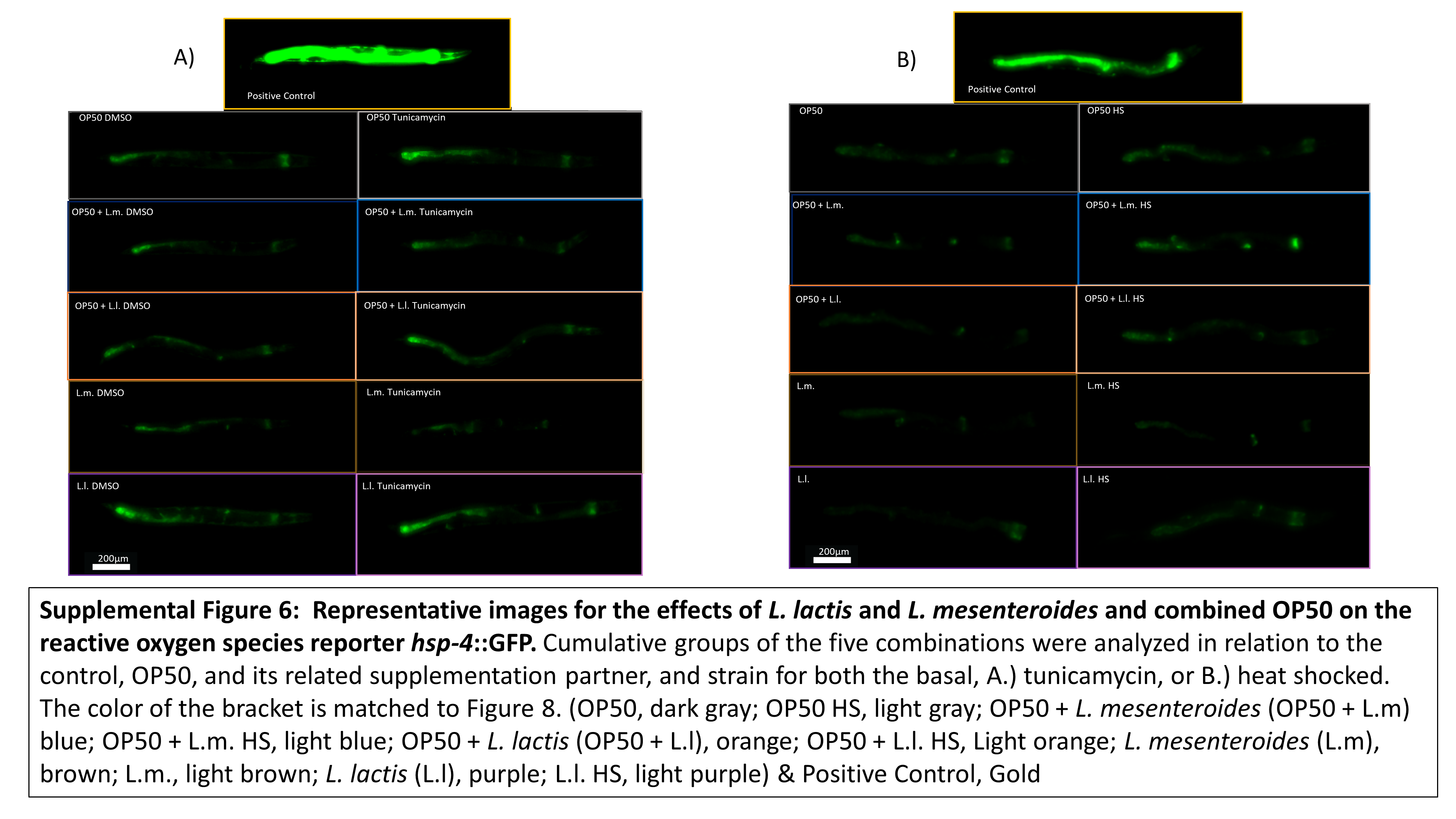

Supplement: Supplementary file 2 [file Image6.tif]

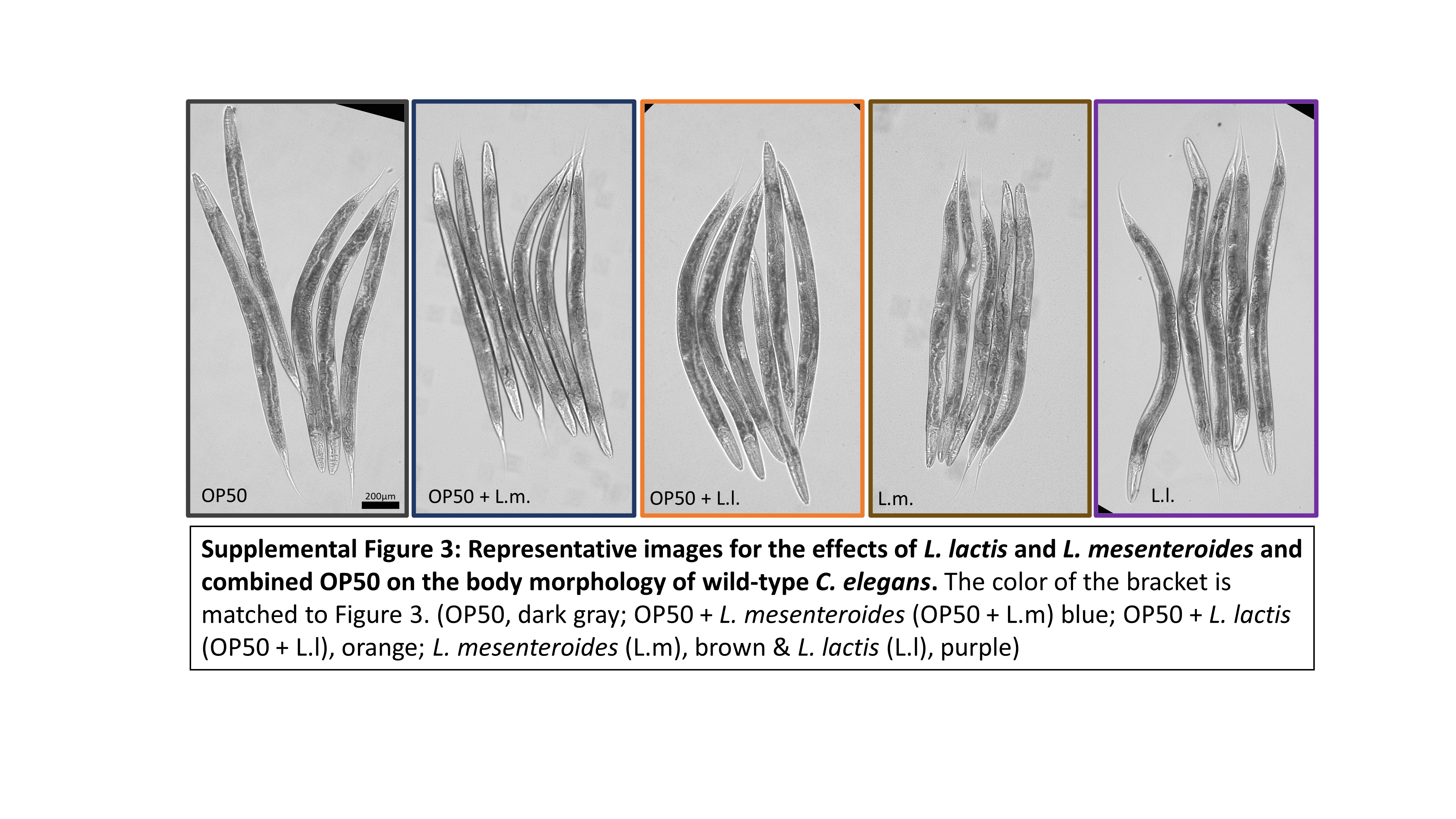

Supplement: Supplementary file 3 [file Image3.TIF]

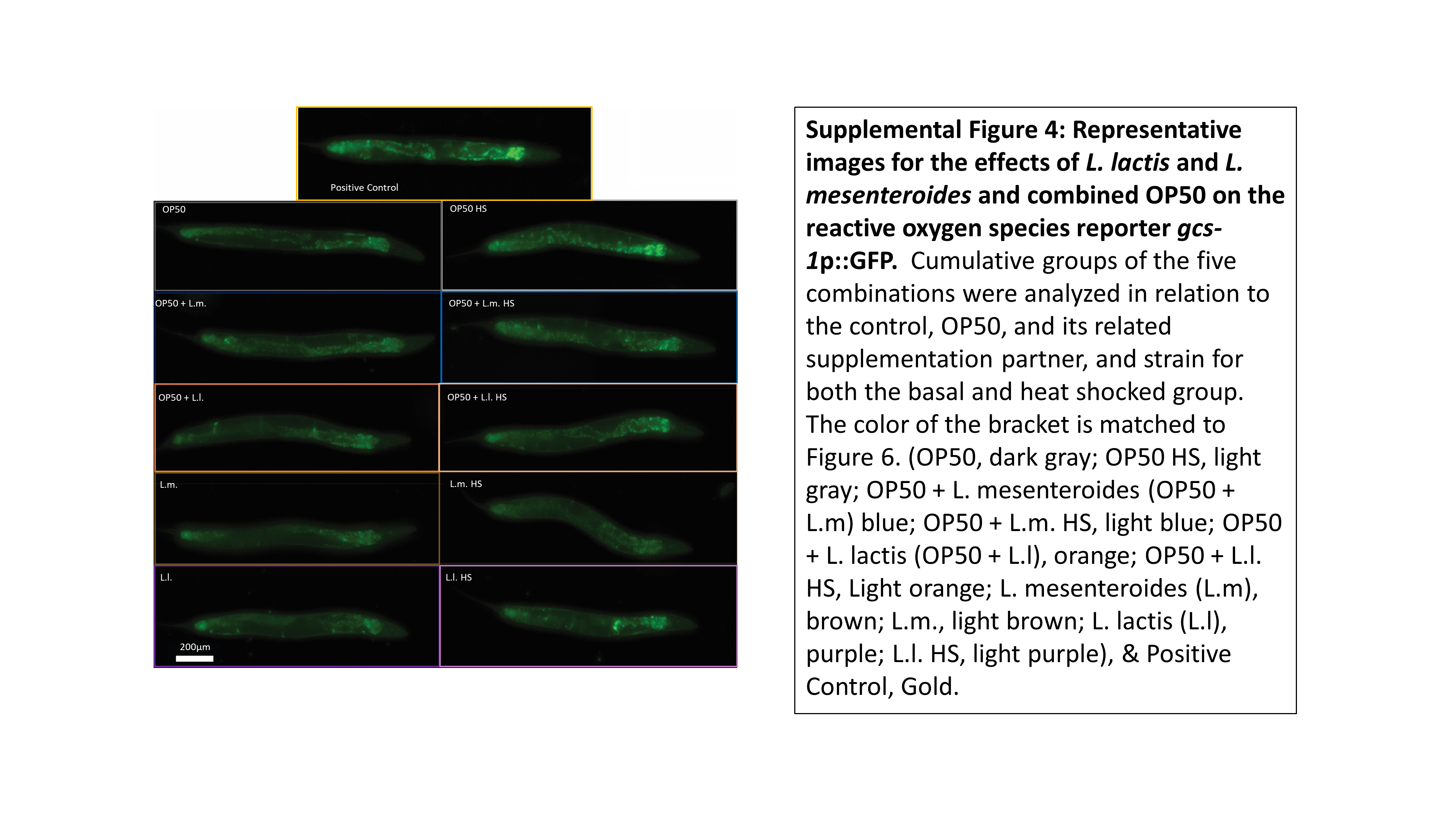

Supplement: Supplementary file 4 [file Image4.TIF]

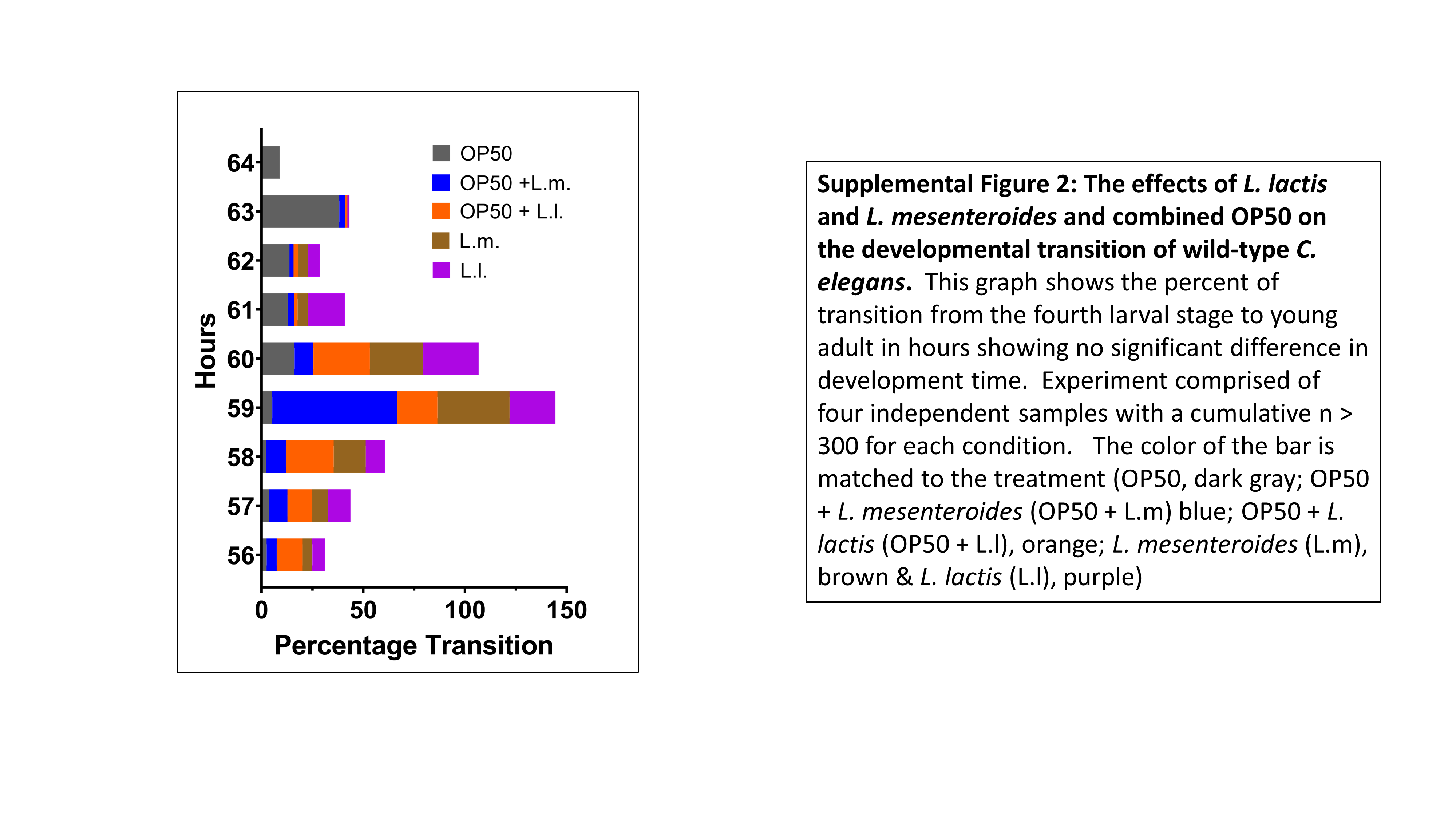

Supplement: Supplementary file 5 [file Image2.TIF]

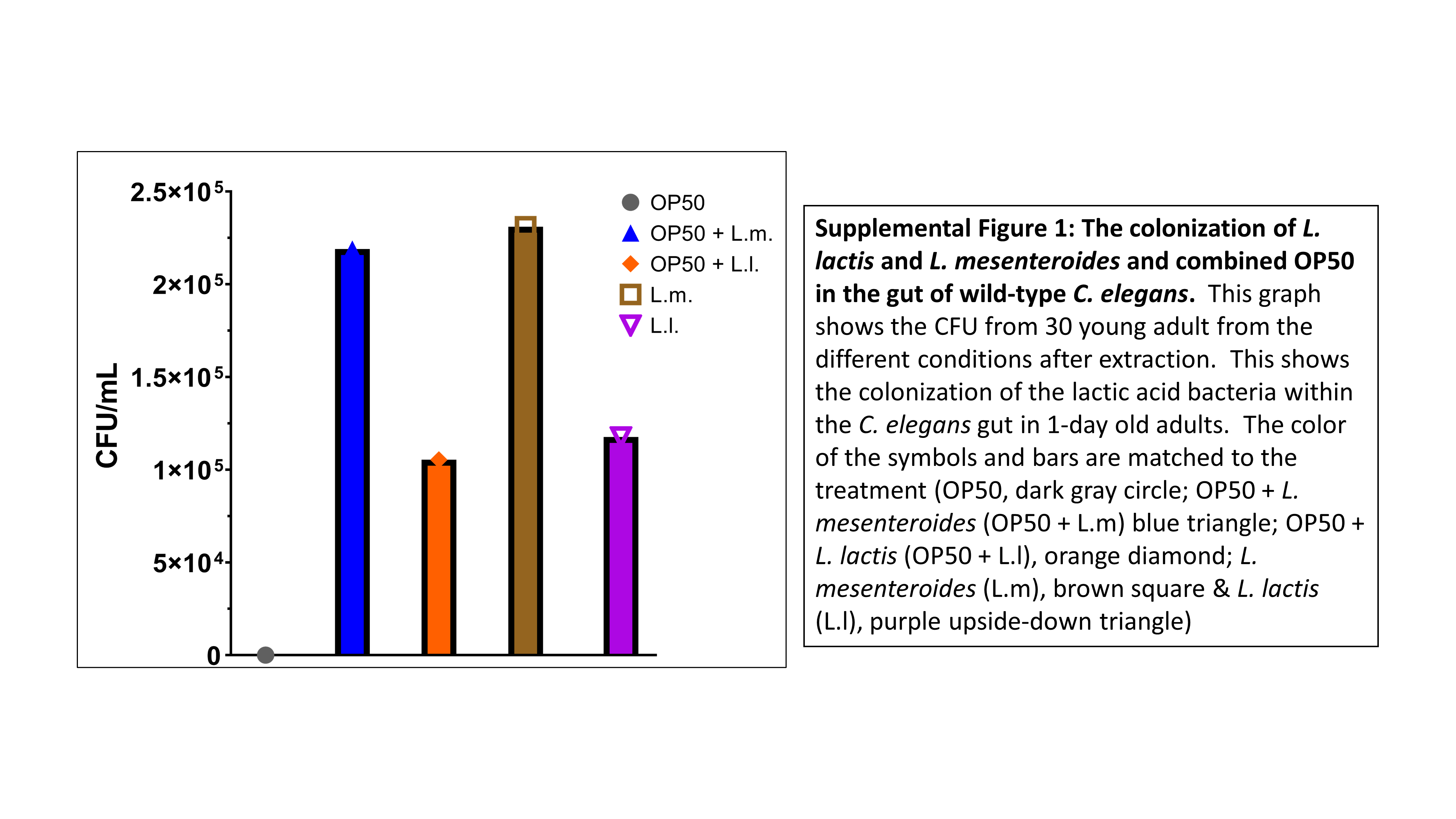

Supplement: Supplementary file 6 [file Image1.TIF]

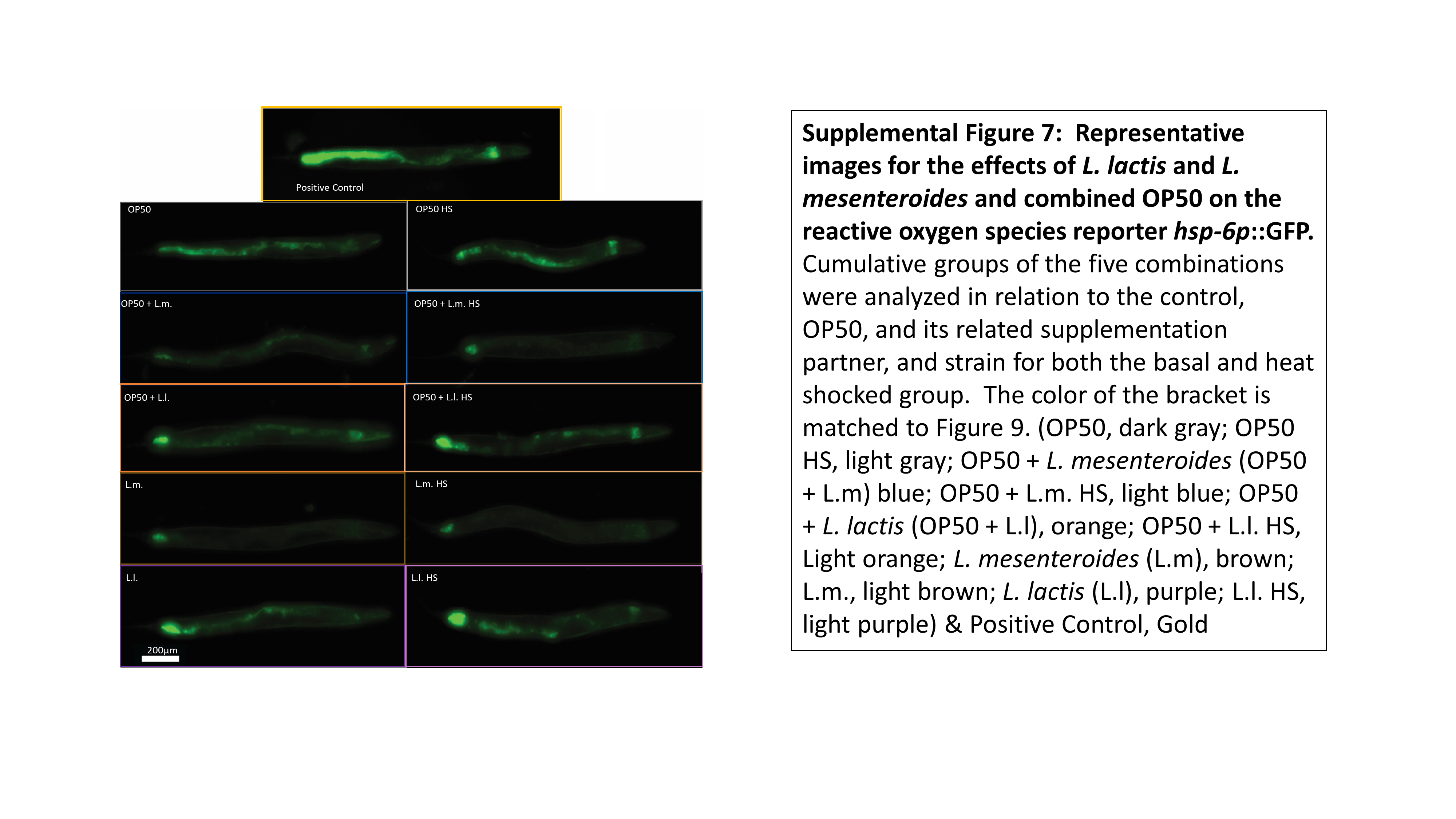

Supplement: Supplementary file 8 [file Image7.TIF]

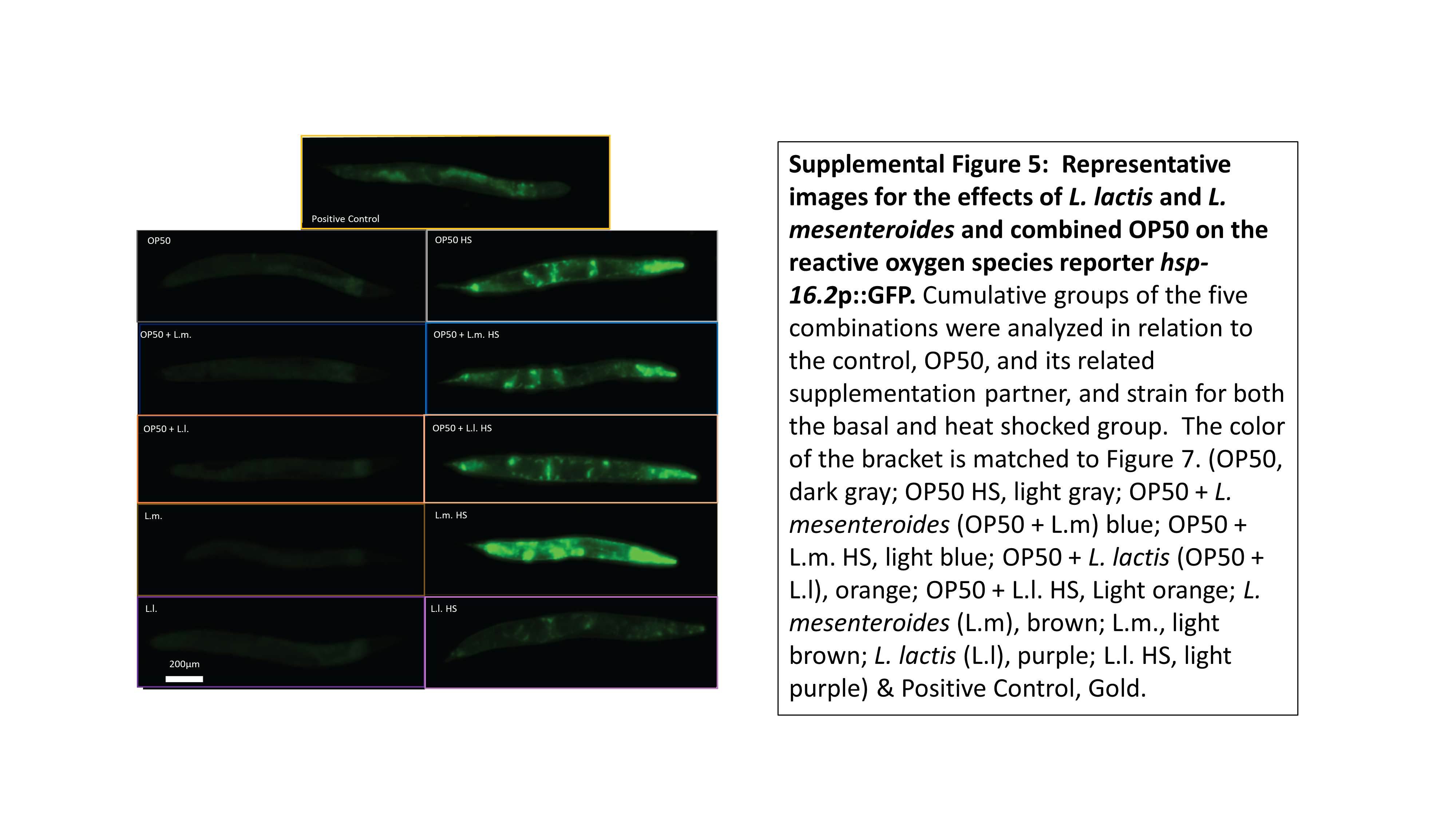

Supplement: Supplementary file 10 [file Image5.TIF]
