# Supplementary material for: Probiotic effects of Lactococcus lactis and Leuconostoc mesenteroides on stress and longevity in Caenorhabditis elegans
Source: Front Physiol. 2023 Sep 12;14:1207705. doi: 10.3389/fphys.2023.1207705 (PMC10522913; doi:10.3389/fphys.2023.1207705)
Supplement: Supplementary file 7 [file DataSheet3.PDF]

Strain Name	Genius/Species	Source	Isolation	CeMbio %	CeMbio + L.m %	CeMbio + L.l %	OP50 + L.m %	OP50 + L.l %
OP50	*E. coli*	CGC	*C. elegans* Labartory Strain	0	0	0	90	90
CEN2ent1	*Enterobacter xiangfangensis*	CGC	Isolate from soil of mesocosm experiment.	12.7	12.7	12.7	0	0
JUb66	*Lelliottia amnigena*	CGC	Natural isolate	12.7	12.7	12.7	0	0
MYb10	*Acinetobacter guillouiae*	CGC	Natural isolate	11.3	11.3	11.3	0	0
JUb19	*Stenotrophomonas maltophilia*	CGC	Natural isolate	10.6	10.6	10.6	0	0
JUb134	*Sphingomonas molluscorum*	CGC	Natural isolate	9.4	9.4	9.4	0	0
MYb11	*Pseudomonas lurida*	CGC	Natural isolate	5.4	5.4	5.4	0	0
MSPm1	*Pseudomonas mendocina*	CGC	Isolate from a *C. elegans* population in a soil, compost laboratory environment.	3.6	3.6	3.6	0	0
BIGb0172	*Comamonas piscis*	CGC	Natural isolate	3.4	3.4	3.4	0	0
BIGb0393	*Pantoea sp.*	CGC	Natural isolate	3.1	3.1	3.1	0	0
MYb71	*Ochrobactrum pecoris*	CGC	Natural isolate	2.1	2.1	2.1	0	0
BIGb0170	*Sphingobacterium sp*	CGC	Natural isolate	1.1	1.1	1.1	0	0
JUb44	*Chryseobacterium sp.*	CGC	Natural isolate	0.8	0.8	0.8	0	0
L.m	*Leuconostoc mesenteroides*	OSU-CHS	Organic Basil	0	10	0	10	0
L.l	*Lactococcus lactis*	OSU-CHS	Organic Basil	0	0	10	0	10
