# Supplementary material for: Probiotic effects of Lactococcus lactis and Leuconostoc mesenteroides on stress and longevity in Caenorhabditis elegans
Source: Front Physiol. 2023 Sep 12;14:1207705. doi: 10.3389/fphys.2023.1207705 (PMC10522913; doi:10.3389/fphys.2023.1207705)
Supplement: Supplementary file 9 [file DataSheet1.PDF]

Supplemental Protocols:

Developmental Transition:

To assess the influence The effects of *L. lactis* and *L. mesenteroides* and combined OP50 on the developmental transition of wild-type C. elegans, nematodes were raised on monocultures and combined lawns. Worms were synchronized through an egg lay protocol on their respective bacterial lawn. The number of adults transition from L4 to young adult (through the appearance of a vulva) over time were scored on an hourly basis at 56-65 hours post egg lay (Dirksen et al. 2020).

Colonization Assay

Modified from Dirksen et al. 2020, we adapted the colonization assay to determine, if there was growth from the monocultures extracted from the gut of the egg lay synchronized worms at the 1-day old adult stage. We counted the lactic acid bacterial colonies after growing overnight at 32°C to determine the CFU values for colonization of the 30 - 1-day old *C. elegans*. The protocol that was largely followed was previously published from Zhang et al. 2020 and is available online on protocols.io (DOI: dx.doi.org/10.17504/protocols.io.rtzd6p6) (Zhang et al. 2020; Dirksen et al. 2020).

References:

Dirksen, Philipp, Adrien Assié, Johannes Zimmermann, Fan Zhang, Adina-Malin Tietje, Sarah Arnaud Marsh, Marie-Anne Félix, et al. 2020. “CeMbio - The Caenorhabditis Elegans Microbiome Resource.” *G3 (Bethesda, Md.)* 10 (9): 3025–39.

Zhang, Fan, Jessica L. Weckhorst, Adrien Assié, Anastasia S. Khodakova, Mario Loeza-Cabrera, Daniela Vidal, and Buck S. Samuel. 2020. “High-Throughput Assessment of Changes in the Caenorhabditis Elegans Gut Microbiome.” *Methods in Molecular Biology* 2144: 131–44.
